# The role of recombinant LH in ovarian stimulation: what’s new?

**DOI:** 10.1186/s12958-025-01361-8

**Published:** 2025-03-10

**Authors:** Carlo Alviggi, Luigi Vigilante, Federica Cariati, Alessandro Conforti, Peter Humaidan

**Affiliations:** 1 Department of Public Health, University of Naples Federico II, Naples, Italy; 2 Department of Neuroscience, Reproductive Science and Odontostomatology, University of Naples Federico II, Via Sergio Pansini, 5, Naples, 80131 Italy; 3 The Fertility Clinic, Skive Regional Hospital, Faculty of Health, Aarhus University, Skive, Denmark

**Keywords:** Luteinizing hormone, Ovarian stimulation, IVF, ART, POSEIDON, Poor responders, Hypo-responders

## Abstract

It is widely recognized that luteinizing hormone (LH) activity is pivotal during folliculogenesis. Nonetheless, the use of LH during ovarian stimulation remains a matter of debate. Indeed, women with good LH function are able to sustain follicle growth and maturation during ovarian stimulation carried out with regimens based on follicle-stimulating hormone (FSH) alone. However, evidence exists that LH activity could be necessary in specific infertile subgroups undergoing assisted reproduction treatment (ART) who are characterized by a functional or constitutive LH deficiency. For instance, women with reduced sensitivity to gonadotropins, also called hypo-responders, usually present with a genetic condition that could impair the function of LH. Furthermore, women of advanced reproductive age present a less functional LH system and consequently reduced androgen production. Reduced ovarian sensitivity and advanced reproductive age represent the main criteria proposed by the POSEIDON group to identify women with impaired prognosis when undergoing ART. Hypogonadotropic hypogonadal women are characterized by undetectable LH levels, thus the addition of LH activity during stimulation is mandatory to achieve satisfactory follicular recruitment. The aim of the present review is to describe the role of recombinant LH in ovarian stimulation, identifying the specific infertile population for whom LH supplementation could improve the outcome of ART.

## Introduction

Luteinizing hormone (LH) plays a crucial role in folliculogenesis. According to the ‘two cell − two gonadotropin’ model [1, 2], LH exerts its activity in theca cells, inducing androgen synthesis, while follicle-stimulating hormone (FSH) induces expression of the aromatase enzyme which, in turn, converts androgens into estrogens. This view has been reconsidered in light of evidence supporting the effect of LH beyond theca cells [3–5]. It was widely recognized that, in granulosa cells, FSH and estrogens induce the expression of LH choriogonadotropin receptor (LHCGR) from the early/mid-follicular phase [1, 6, 7]. During this stage, LH could mimic the actions of FSH on granulosa cells, including the induction of aromatase activity [8, 9]. From a clinical point of view, LH activity could sustain alone follicular growth in the last stage of ovarian stimulation (OS) in women undergoing assisted reproduction independently of FSH [10]. In detail, Filicori et al. observed that women who received only LH in the form of low-dose human chorionic gonadotropin (hCG) in the last part of OS had similar estradiol levels and number of large (> 14 mm) preovulatory follicles than those who received only FSH throughout the stimulation [10]. Furthermore, stimulation with LH alone could significantly reduce the numbers of small antral follicles, thus facilitating selection of large follicles [10]. This study therefore supports the concept that the growth of dominant and larger follicles depends mainly on the activity of a functional LHCGR [7]. The most recent formulations are based on pure LH, which is characterized by more pronounced anti-apoptotic and proliferative effects than hCG [11, 12]. LH could also prevent the apoptosis induced by the cytotoxic effect of chemotherapeutic agents, thereby preserving fertility in in vivo models [13].

In synergy with FSH, LH could promote the production of paracrine factors playing a crucial role in folliculogenesis, such as insulin-like growth factors and inhibin B [5, 14]. LH is also fundamental to ovulation, since it induces protease activity and oocyte maturation [15]. Finally, LH is the main inductor of luteinization, essential for progesterone production and endometrial support during implantation [15].

Recent studies have suggested that LH might have extragonadal actions, as the LHCGR is expressed in the human endometrium [16, 17] and in non-gonadal tissue [18]. However, the relevance of LHCGR in non-gonadal tissue [18–20] is still debated. Although exciting results were obtained in mice [21–23], the existence of human extragonadal gonadotropin receptors would imply an absence of off-target endocrine effects; much of these data should be revised in light of lack of specificity of the detection methods available, artificial experimental settings, and methodological biases [24–29].

The use of LH during OS has been a matter of debate for several years, supported by the fact that medications used to prevent premature ovulation, such as gonadotropin-releasing hormone (GnRH) antagonists and agonists, will cause a transient deficiency of endogenous LH [30–32]. Among women in whom LHCGR-dependent signals are sufficient to support folliculogenesis, the need for LH supplementation appears to be limited during OS [31]. In fact, residual circulating LH, likely to persist during suppression of the pituitary, may be sufficient to sustain LHCGR activity and support follicle growth and maturation during treatment with FSH alone. This effect is observable during routine in vitro fertilization treatment in the general population, who do not require LH supplementation during OS to obtain a successful response to OS [33, 34]. Nonetheless, there is evidence that LH activity could be necessary in specific infertile subgroups undergoing IVF treatment.

The aim of the present review is to describe the role of recombinant LH supplementation during OS, identifying the specific infertile population for whom LH activity supplementation could improve the outcomes of assisted reproduction treatment (ART).

## Methods

This narrative review was conducted through a literature search in PubMed, Scopus, Embase, and the ISI Web of Science database. The following search terms were used: ‘LH’, ‘recombinant LH’, luteinizing hormone’, ‘ovarian stimulation’, ‘ART’, ‘IVF’, ‘ovarian stimulation’, and ‘ovulation induction’. We mainly included clinical studies that analyzed the impact of LH on controlled OS, dating from the inception of the database used to June 2023 (Table 1). No language restriction was adopted.

**Table 1:** Key clinical articles included in the review

	Study design	Patients involved	Intervention	Comparator	Results
**Hypo-responders**					
Conforti et al. 2019	Meta-analysis (4 RCTs + 1 prospective study)	Hypo-responders	r-hFSH and r-hLH co-treatment	r-hFSH monotherapy	Significantly higher clinical pregnancy rates, implantation rates, number of oocytes retrieved
**Advanced maternal age**					
Conforti et al. 2021	Meta-analysis (12 RCTs)	Advanced maternal age (≥35 years)	r-hFSH and r-hLH co-treatment	r-hFSH monotherapy	Significantly higher clinical pregnancy rates, and implantation rates, in women between 35–40 years old
**Hypogonadotropic hypogonadal**					
Carone et al. 2012 [94]	RCT	Hypogonadotropic hypogonadal women	r-hFSH and r-hLH co-treatment	hMG	Significantly higher clinical pregnancy rate
Huseyin et al. 2019 [99]	Retrospective study	Hypogonadotropic hypogonadal women	r-hFSH and r-hLH co-treatment	hMG	Significantly lower cancellation rate
**Progesterone rise**					
Werner et al. 2014 [87]	Retrospective study	10,280 patients first IVF cycle	FSH plus LH formulations	FSH monotherapy	Stimulations using no administered LH had the highest risk of progesterone elevation ≥ 1.5 ng/mL
Hugues et al. 2012 [91]	Systematic review (34 studies)	Women undergoing MAR techniques	hMG, r-hLH, or hCG	FSH monotherapy	Comparable risk of progesterone rise
**Implantation failure**					
Rahman et al. 2017 [92]	RCT	Women with 2 previous failed implantations	r-hFSH and r-hLH co-treatment	r-hFSH monotherapy	Significantly higher implantation rate and pregnancy rate; significantly lower abortion rate
**Poor response according to Bologna criteria**					
Humaidan et al. 2017 [42]	RCT	Women fulfilling Bologna criteria	r-hFSH and r-hLH co-treatment	r-hFSH monotherapy	Significantly higher cumulative live birth rate in women with moderate or severe BSC
Lehert et al. 2021 [44]	Retrospective study	Women fulfilling Bologna criteria	r-hFSH and r-hLH co-treatment	r-hFSH monotherapy	Significantly higher cumulative live birth rate in women with moderate or severe BSC

*BSC* baseline severity score, *FSH* follicle-stimulating hormone, *hCG* human chorionic gonadotropin, *hMG* human menopausal gonadotropin, *LH* luteinizing hormone, *MAR* medically assisted reproduction, *RCT* randomized controlled trial, *r-hFSH* recombinant human FSH, *r-hLH* recombinant human LH

## Results and discussion 

### LH in women with poor ovarian response to stimulation according to the ESHRE/Bologna criteria

The ESHRE/Bologna criteria are widely adopted to identify women with poor response to OS [35]. These criteria are based on the presence of at least two of the following characteristics: (i) advanced maternal age (> 40 years) or any other poor ovarian response risk factor; or (ii) a previous episode of poor ovarian response (POR; defined as ≤ 3 oocytes retrieved after a conventional stimulation dose), a low ovarian reserve test in terms of anti-Müllerian hormone (AMH), and a low antral follicle count (AFC). These criteria have been debated, because they risk classifying a very heterogeneous group of women with a different reproductive prognosis following IVF treatment [36–39]. Indeed, in a large retrospective study (*N* = 821 women), live birth rates were significantly different in various subgroups of poor ovarian responders fulfilling the Bologna criteria, with the most favorable outcome in younger women (ages < 40 years) [40]. Similarly, Romito et al. (2020) observed that the cumulative live birth rate was statistically significantly different among subgroups of poor ovarian responders classified by the Bologna criteria [41]. So far, the largest multicenter randomized controlled trial (RCT) to explore the use of recombinant human LH (r-hLH) in poor ovarian responders aligned with the Bologna criteria was conducted by Humaidan et al. (2017) [42]. A total of 939 women undergoing IVF treatment were randomized to receive OS with recombinant human FSH (r-hFSH) and r-hLH (300 and 150 IU, respectively) from day 1 of stimulation or r-hFSH alone (300 IU daily). All women underwent a long GnRH agonist down-regulation protocol.

In the total population, no differences were observed between the two groups of treated patients regarding implantation rate or live birth rate. However, a post-hoc analysis revealed that r-hFSH and r-hLH co-treatment actually had a positive effect on the live birth rate of specific subgroups of women fulfilling the Bologna criteria. Thus, the study population was stratified into three different groups, adopting a so-called Baseline Severity Score (BSC). This score was based on the following characteristics: (i) age ≥ 40; (ii) reduced ovarian reserve (AMH < 0.5 ng/mL) or < 2 oocytes retrieved during the most recent ART cycle. The BSC for a subject could reach the value of 0 (mild) if none of these criteria were met; 1 (moderate) if one criterion was met; or 2 (severe) if two criteria were met. Interestingly, women with moderate or severe BSC had a higher live birth rate when supplemented with r-hLH compared to patients treated with r-hFSH alone. Conversely, women with a mild BSC had a higher live birth rate when stimulated with r-hFSH alone, compared to patients supplemented with r-hLH. Subsequently, the BSC score was ‘renamed’ as the Poor Responder Outcome Prediction (PROsPeR) score, which showed good discrimination to predict the live birth rate of women fulfilling the Bologna criteria [43, 44]. Recently, in a large retrospective real-world analysis of 9,787 IVF treatments, Lehert et al. (2021) confirmed that the cumulative live birth rate (defined as the occurrence of live birth per started controlled OS further to transfer of fresh and frozen embryos generated from the same OS) was significantly higher in patients with a moderate or severe PROsPeR score who received r-hFSH and r-hLH co-treatment, compared with moderate or severe PROsPeR score patients who received r-hFSH monotherapy [45].

In conclusion, r-hLH supplementation could improve the live birth and cumulative live birth rates in specific subgroups of women fulfilling the Bologna criteria. In detail, women with the highest PROsPeR score are likely to benefit most from r-hLH supplementation during OS.

#### LH in hypo-responders (POSEIDON groups 1 and 2)

The hypo-responsive patient is characterized by a reduced sensitivity to exogenous gonadotropins during IVF treatment [46], displaying a discrepancy between the AFC at the beginning of stimulation and the number of preovulatory follicles and oocytes retrieved after stimulation [46]. These patients often have a stagnation in follicular growth during OS, especially when undergoing a long GnRH protocol or a few days after the introduction of the GnRH antagonist [47, 48]. Compared with normal responders, hypo-responders have a lower chance of ART success, and were therefore included in the POSEIDON criteria. Indeed, POSEIDON groups 1 and 2 show a reduced ovarian response to OS, with a suboptimal (4–9 oocytes) or poor (≤ 3 oocytes) retrieval of oocytes despite an adequate ovarian reserve (AMH > 1.2 ng/mL or AFC > 5) [39, 49, 50].

So far, three RCTs and one prospective cohort study have investigated the effect of LH supplementation in women with a hypo-response profile [12, 47, 48, 51]. All women included in these studies underwent a long GnRH agonist down-regulation protocol and experienced follicular stagnation during OS. A recent meta-analysis performed on those studies concluded that the addition of r-hLH during OS lead to a significant increase in oocyte number, implantation rate, and clinical pregnancy rate [52]. Only the RCT conducted by Ferraretti et al. (2004) reported data about live birth rate per cycle, confirming an increased live birth rate in hypo-responders undergoing stimulation with r-hFSH and r-hLH co-treatment compared with those who received r-hFSH alone during their OS [47]. Notably, these researchers also observed a significantly higher pregnancy rate in hypo-responders who underwent r-hFSH and r-hLH co-treatment compared to those who received combination therapy with r-hFSH and human menopausal gonadotropin (hMG).

The reason behind the need to add LH activity in women affected by hypo-responsiveness is still not fully clear, but it has been hypothesized that women with hypo-response have a relative LH deficiency [31]. In other words, in the hypo-responder patient, endogenous LH levels are insufficient to secure appropriate stimulation when the pituitary axis is suppressed [32]. The LH relative deficiency that characterizes women with hypo-response could be linked to specific genetic variants, affecting the LH system. Indeed, GnRH agonist down-regulated women who had a specific genetic variant of the LH β chain showed a typical hypo-response profile during OS with exogenous FSH and required higher consumption of FSH during OS [53, 54]. More recently, Ku at al. (2021) observed that the variant LH β gene was associated with a lower clinical pregnancy rate in GnRH antagonist cycles, but not in long GnRH agonist down-regulated cycles [55]. This observation is consistent with several lines of evidence suggesting that endogenous LH levels are more suppressed during GnRH antagonist treatment compared with the GnRH agonist down-regulated cycle [56–58].

Finally, women who express a common variant of the LHCGR receptor also seem to benefit from exogenous LH during OS [59, 60]. Hypo-response to monotherapy with FSH might be linked to the paracrine activity exerted by exogenous LH in women with hypo-response. In detail, r-hLH supplementation in women with hypo-response could modify the follicular fluid steroid composition, changing it to a more physiologic composition in terms of estrogens and progestins [61, 62]. This finding emerges from a prospective analysis of 111 follicular fluid samples obtained from women with hypo-response who underwent r-hFSH/r-hLH co-treatment versus r-hFSH monotherapy [62].

The appropriate dosage and timing of r-hLH in women with hypo-response is still a matter of debate. In women undergoing the long GnRH agonist down-regulation protocol, it seems that r-hLH should be supplemented once follicular stagnation is observed, typically between days 7 and 10 of OS (estradiol levels < 180 pg/mL, no follicles > 10 mm diameter) [47, 48]. Regarding the dosage, hypo-responders supplemented with 150 IU of r-hLH daily (‘rescue protocol’) had a significantly higher number of oocytes and mature oocytes retrieved compared to hypo-responders who received 75 IU r-LH daily. So far, very few studies and no RCTs have investigated the effect of r-hLH supplementation in women with hypo-response who were co-treated with a GnRH antagonist; however, the follicular stagnation pattern is the same and is usually seen 2–3 days after starting GnRH antagonist co-treatment.

In conclusion, women with hypo-response seem to benefit from r-hLH supplementation during OS; the most appropriate dosage and timing in women undergoing a long GnRH agonist down-regulation regimen seems to be 150 IU/day r-LH, starting from the day that follicular stagnation is detected.

#### LH in women of advanced reproductive age (POSEIDON groups 2 and 4)

Advanced reproductive age is characterized by a reduced reproductive prognosis in ART [39, 63]. A more pronounced decline is observed after 35 years of age, reflecting a gradual decrease in ovarian reserve and oocyte quality [64, 65]. Indeed, the IVF success rate decreases dramatically, to < 5%, beyond the age of 43/44 years [63]. Chronological age is generally considered one of the main parameters to predict the prognosis of ART. In contrast to the Bologna criteria, the POSEIDON group suggested 35 years as the most appropriate cut-off and identified four different segments of prognosis based on age. Advanced-age women with a good ovarian reserve were stratified into POSEIDON group 2, whereas those with a poor ovarian reserve were stratified into POSEIDON group 4, with a distinct significant difference in reproductive outcomes between groups [38, 66, 67].

Aneuploidy rates of human embryos are probably the most relevant cause of the decrease in IVF success rate in advanced-age women [68]. In a recent study, it was demonstrated that the probability of having a euploid embryo decreases from 24.5 to 1.2% in women aged 28–44 years [69]. Apart from embryo quality, several lines of research have suggested that advanced reproductive age is also associated with relative LH deficiency [31, 70]. This hypothesis is supported by the fact that LH-related androgen production dramatically decreases in advanced age women [71–73]. Although it has been hypothesized that the androgen deficiency seen in the aging woman could be compensated by androgen supplementation [74], some researchers have suggested that exogenous LH supplementation would more optimally induce local follicular androgen production compared to exogenous androgen supplementation [75, 76].

From a clinical point of view, several RCTs have been reported comparing r-hFSH and r-hLH co-treatment versus r-hFSH monotherapy in women of advanced age [77–79]. A recent meta-analysis of RCTs concluded that r-hFSH/r-hLH co-treatment during OS significantly benefited women between the age of 35 and 40 years, in terms of implantation (OR 1.49, CI 95% 1.10–2.01; *p* = 0.01) and clinical pregnancy rate (OR 1.45, CI 95% 1.05-2.00; *p* = 0.03), whereas no difference was seen in higher-age groups [80]. The lack of effect beyond 40 years of age could be masked by the impact of age-related aneuploidy rates on IVF prognosis [68, 80].

The most plausible positive effect exerted by exogenous LH supplementation seems to be related to an improvement in oocyte/embryo quality [81]. Thus, the follicular fluid LH level was identified as a good marker of oocyte/embryo competence [82]. In a large age-adjusted RCT, Bosch et al. (2011) observed higher fertilization rates in women aged 35–39 years co-treated with r-hFSH and r-hLH versus r-hFSH monotherapy (68% ± 25% vs. 61.2% ± 27.3%; *p* = 0.027) [78]. In another study, Ruvolo et al. (2007) observed that in women treated with exogenous r-hLH, the apoptosis rate in cumulus cells, expressed as lower rate of chromatin fragmentation (12.1% vs. 18.2%; *p* < 0.05), and number of immature oocytes (0.58 vs. 2.33; *p* < 0.01) were significantly reduced compared to women treated with r-hFSH alone [12].

In conclusion, LH activity could be proposed in advanced-age women undergoing OS; however, a significant effect in terms of implantation and clinical pregnancy was only seen in women between 35 and 40 years of age.

#### LH in women with progesterone rise and impaired embryo implantation

Apart from a positive effect on oocyte/embryo quality, the improved effect of LH supplementation on implantation might also be explained by an LH-mediated effect on the endometrium [83]. Thus, LH is able to modulate several factors that play a role during embryo implantation, such as colony-stimulating factor-1, cytokine leukemia inhibiting factor, glycodelin, interleukin-1, integrins, and mucin 1 [84].

Another possible benefit exerted by LH activity during OS is the potential reduction in late follicular serum progesterone levels, which by retrospective analyses has been suggested to be associated with lower ongoing implantation and pregnancy rates in fresh embryo transfer IVF cycles [85, 86]. Thus, in an analysis of 10,280 patients undergoing their first IVF cycle, OS without LH activity (hMG) resulted in a significantly higher risk of late follicular progesterone rise [87]. In the same line, the MERIT trials randomly assigned women to receive either r-hFSH or hMG alone showed significantly higher late follicular progesterone levels in patients treated with r-hFSH compared to those receiving hMG-only protocols [88]. This finding could be explained by the fact that progesterone rise is mainly driven by high FSH dosing during OS [89]. Conversely, by suppressing the development of small follicles, LH activity could reduce progesterone levels [85, 90]. However, a systematic review of 34 studies did not confirm that LH activity could a have a modulating effect on serum progesterone levels [91]. Indeed, the authors observed a decrease in serum progesterone only when LH was prescribed from the beginning of OS; this makes sense from a physiological point of view, as this is the time point to suppress the growth of smaller follicles [91].

To date, only one study has explored the effect of exogenous LH supplementation in women with a history of implantation failure [92]. In that study, 61 women with a history of two failed embryo transfers who underwent OS, co-treated with a flexible GnRH antagonist protocol, were randomized into two groups: the study group, which was supplemented with r-hLH from the day of GnRH antagonist co-administration (*n* = 29); and the control group (*n* = 32), which was stimulated with r-hFSH alone. Interestingly, the implantation (19% vs. 9%; *p* < 0.01) and positive pregnancy test (48.3% vs. 25%; *p* < 0.03) were significantly higher in the study group versus control group; moreover, the pregnancy loss rate was significantly lower in the study group versus control group (21% vs. 37.5%; *p* < 0.01).

Despite these promising findings, the evidence collected so far is not sufficient to recommend the use of r-hLH in women with a history of implantation failure. As for late follicular progesterone rises and r-hLH supplementation, conflicting evidence exists; but the effect of LH activity appears to be more pronounced when LH is administered from day 1 of OS.

#### LH in hypogonadotropic hypogonadal women

In women with hypogonadotropic hypogonadism (HH) with very low gonadotropin levels (World Health Organization group I anovulatory women), the accumulated evidence so far supports the need for LH activity during OS [93, 94]. Indeed, r-hFSH stimulation alone cannot support follicular growth in women with HH [93, 95, 96]. In other words, HH women do not have sufficient circulating endogenous LH levels to support optimal follicular development. Both human menopausal gonadotropin (hMG) and r-FSH with r-hLH, compared to r-hFSH alone, resulted in a significantly higher ovarian response to OS in HH women [97, 98]. In a single RCT, 35 women with HH were randomized to receive 150 IU of highly purified hMG (*n* = 18) or 150 IU r-hFSH plus 75 IU r-hLH daily for a maximum of 16 days (*n* = 17) [94]. Women stimulated with r-hLH had significantly higher pregnancy rates compared with those who underwent hMG stimulation (55.6% vs. 23.3%; *p* = 0.01) [94]. Nonetheless, ovulation induction was similar between groups.

In a more recent retrospective analysis involving a total of 99 HH women undergoing OS and intrauterine insemination, similar pregnancy rates were observed comparing women treated with r-hFSH and r-hLH versus those who received hMG for OS. Nonetheless, the cancellation rates were significantly higher in women who underwent hMG stimulation versus those co-treated with r-hLH (29% vs. 8.1%; *p* < 0.05) [99]. Taken together, despite being more costly, r-hLH supplementation in the HH woman seems to result in a better reproductive outcome compared to hMG.

## Knowledge gaps and future research

The evidence so far collected suggests that LH supplementation could be considered in specific IVF women. Nonetheless, further analysis is required in larger populations with specific prognostic classifications based on POSEIDON criteria. Furthermore, few studies have investigated the effect of LH in hypo-responder women undergoing antagonist protocols. Another interesting field for future research is the growing role of r-hLH in promoting AFC recruitment [100] and fertility preservation [13]; despite promising findings, more study data are required before any clinical conclusions can be drawn.

## Conclusion

**Fig. 1:**
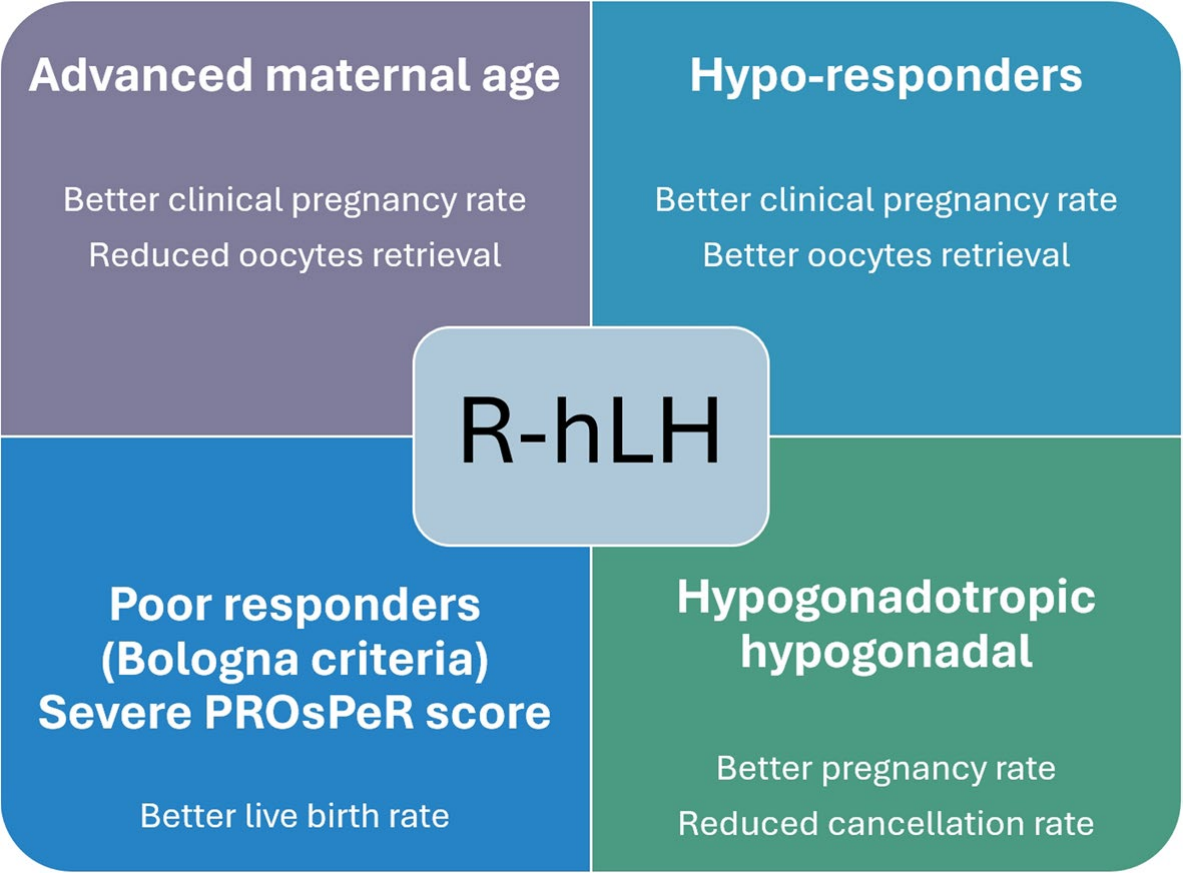
Main patient populations benefiting from LH supplementation, and the key mechanisms involved

In conclusion, this literature search on the effect of r-hLH in specific - and LH activity (HMG) in general - supports the use of r-hLH for OS in the following sub-groups of IVF patients: (i) the POR patient aligned with the Bologna POR criteria with the highest PROsPeR score; (ii) the hypo-responder to r-hFSH monotherapy (POSEIDON groups 1 and 2); (iii) patients of advanced reproductive age up to and including 40 years of age (POSEIDON groups 2 and 4); and (iv) the hypogonadotropic hypogonadal patient (Fig. 1). For all subgroups to obtain the most optimal benefit, r-hLH supplementation should commence from day 1 of ovarian stimulation. Whether further patient populations might benefit from supplementation with r-hLH needs to be explored in future trials and analyses.

## Data Availability

Not applicable.

## Abbreviations

AFC: antral follicle count
AMH: anti-Müllerian hormone
ART: assisted reproduction treatment
BSC: Baseline Severity Score
FSH: follicle-stimulating hormone
GnRH: gonadotropin-releasing hormone
hCG: human chorionic gonadotropin
HH: hypogonadotropic hypogonadism
hMG: human menopausal gonadotropin
LH: luteinizing hormone
LHCGR: luteinizing hormone choriogonadotropin receptor
OS: ovarian stimulation
POR: poor ovarian response
RCT: randomized controlled trial
